# Patterns of community assembly in the developing chicken microbiome reveal rapid primary succession

**DOI:** 10.1002/mbo3.821

**Published:** 2019-03-03

**Authors:** Stephanie D. Jurburg, Michael S. M. Brouwer, Daniela Ceccarelli, Jeanet van der Goot, Alfons J. M. Jansman, Alex Bossers

**Affiliations:** ^1^ Department of Infection Biology Wageningen Bioveterinary Research Lelystad The Netherlands; ^2^ German Centre for Integrative Biodiversity Research iDiv (Halle/Jena/Leipzig) Leipzig Germany; ^3^ Department of Bacteriology and Epidemiology Wageningen Bioveterinary Research Lelystad The Netherlands; ^4^ Wageningen Livestock Research Wageningen The Netherlands

**Keywords:** broiler, chicken microbiome, community assembly, fecal microbiome, primary succession

## Abstract

The fine‐scale temporal dynamics of the chicken gut microbiome are unexplored, but thought to be critical for chicken health and productivity. Here, we monitored the fecal microbiome of healthy chickens on days 1–7, 10, 14, 21, 28, and 35 after hatching, and performed 16S rRNA amplicon sequencing in order to obtain a high‐resolution census of the fecal microbiome over time. In the period studied, the fecal microbiomes of the developing chickens showed a linear‐log increase in community richness and consistent shifts in community composition. Three successional stages were detected: the first stage was dominated by vertically transmitted or rapidly colonizing taxa including *Streptococcus* and *Escherichia/Shigella*; in the second stage beginning on day 4, these taxa were displaced by rapid‐growing taxa including *Lachnospiraceae* and *Ruminococcus‐*like species variants; and in the third stage, starting on day 10, slow‐growing, specialist taxa including *Candidatus Arthrobacter* and *Romboutsia* were detected. The patterns of displacement and the previously reported ecological characteristics of many of the dominant taxa observed suggest that resource competition plays an important role in regulating successional dynamics in the developing chicken gut. We propose that the boundaries between successional stages (3–4 and 14–21 days after hatching) may be optimal times for microbiome interventions.

## 1 INTRODUCTION

The last half century has seen poultry production in the world grow more than fivefold, and this trend is expected to continue (FAO, 2016). Poultry is the world's primary source of animal protein (FAO, 2018); the global population of chicken exceeds 40 billion individuals (Oakley, Lillehoj, et al., 2014). Broiler chicken meat comprises the majority of the poultry meat industry, and ensuring the health and productivity of broilers has become a key aspect of this industry (Ballou et al., 2016). Recent studies using high throughput sequencing, have shown the role of the gastrointestinal tract (GIT) microbiome in gastrointestinal development, nutrient absorption, and pathogen invasion resistance (Crhanova et al., 2011; Pan & Yu, 2013; Park et al., 2013; Pedroso & Lee, 2015; Yeoman et al., 2012). Concurrently, initiatives to control antibiotic use in production animals, especially the restriction in use of growth promoters worldwide to prevent the emergence of antibiotic resistance has made the manipulation of intestinal microbiomes in poultry an attractive alternative to support innate immunity and improve health (Kogut & Arsenault, 2016; Pedroso & Lee, 2015), as well as productivity (Singh et al., 2014). Supplementing the diets of developing broilers with probiotics or applying vaccines have long‐term effects on the broilers’ GIT microbiome composition (Ballou et al., 2016). In particular, feed additives have altered microbial activity in the GIT as well as disease susceptibility (Engberg, Hedemann, Steenfeldt, & Jensen, 2004; Mitsch et al., 2004; Owens, Tucker, Collins, & McCracken, 2008), but the exact mechanisms responsible for these effects remain unclear (Oakley, Lillehoj, et al., 2014).

The chicken GIT microbiome is most susceptible to interventions in early life, and is supported by the *competitive exclusion* principle, which refers to the observation that pathogens are less successful in colonizing the chicken GIT when their native microbiomes are more diverse, later in life (Nurmi & Rantala, 1973). The proposed mechanism for this is direct or indirect competition for resources in the GIT with the native microbes, resulting in a lack of available niche space (Nurmi & Rantala, 1973). This concept was developed from studies which found that broilers were most vulnerable to *Salmonella infantis* during the first week after hatching, and that the oral administration of a mixed bacterial culture derived from the GIT of adult chickens resulted in prevention of colonization by *S. infantis* in young broilers (Nurmi & Rantala, 1973; Rantala & Nurmi, 1973). In parallel, research into the potential of probiotic therapies, as live microbial feed supplements, to alter the chicken GIT microbiome overwhelmingly finds that they are most effective when applied during early life (Ballou et al., 2016; Oakley, Lillehoj, et al., 2014).

Available studies show that during early development, the chicken GIT microbiome undergoes rapid changes, and represents a successional landscape which is gradually colonized by bacteria over time (Lu et al., 2003; Oakley, Buhr, et al., 2014; Oakley & Kogut, 2016). A weekly sampling of broilers showed large shifts in the cecal and fecal microbiomes each week as well as a gradual increase in community complexity (Oakley & Kogut, 2016). Another study found the cecal and ileal communities of 3‐day‐old broilers to be distinct from subsequent samples taken at a weekly interval (Lu et al., 2003); and yet another study showed major shifts in the cecal microbiome between the first and third days after hatching (Ballou et al., 2016). From the time of hatching, commercial broilers are exposed to a wide range of sources of microorganisms, being those in the environment, litter, water, and feed, may colonize the broiler GIT in the first weeks of life (Pedroso & Lee, 2015). In addition, the broiler GIT undergoes developmental changes which affect the microbiome as the different segments of the GIT become differentiated. In one case, the microbiomes of the cecum and ileum in broilers were not different until 14 days after hatching, although current methods are more sensitive (Lu et al., 2003). In another study, the immune system developed concurrently with the microbiome (Crhanova et al., 2011). Bacterial community succession occurs rapidly, and experiments sampling successional microbial systems at a high temporal resolution find that during early colonization, the microbial community may exhibit radical shifts in composition at a daily scale (Cong et al., 2017; Gilbert et al., 2010), in correspondence with bacterial growth cycles and their ecological niches (Barnard, Osborne, & Firestone, 2015). Defining microbial succession in the GIT is critical to understanding gut community assembly, disturbance responses, and disease (Marino, Baxter, Huffnagle, Petrosino, & Schloss, 2014), but they have not been studied at a sufficient temporal resolution to understand the mechanisms behind the observed dynamics, particularly during the first week of development.

Here, we used 16S rRNA gene sequencing to monitor the fecal microbiome of broilers from hatch to 35 days after hatching. We focused on the fecal microbiome as a proxy for the GIT microbial development because of the ease of sampling for high temporal resolutions, and because we were interested in successional dynamics in the GIT rather than the exact composition of a specific section of the GIT microbiome. In order to constrain primary succession, we sampled the broilers daily for the first week, when the most rapid dynamics were expected, and then weekly for the duration of the experiment. Our aims were (a) to reveal the temporal dynamics of microbial colonization of the broiler GIT, and (b) to contribute further resolution and mechanistic insight into the susceptibility of the GIT microbiome to interventions in early life, as applied to broilers.

## 2 MATERIALS AND METHODS

### 2.1 Sample collection

Day‐old broilers (strain Cobb 308, *n* = 14) were received from a commercial hatchery (Day 0, 1–24 hr after hatching) and housed in a litter‐covered floor pen (wood shavings, 1.5 m^2^) thereafter. The study employed only male birds to exclude between‐individual variability arising from sex. The birds received* ad libitum* a starter, grower, and finisher diet over days 0–13, 14–27, and 28–42, respectively. The nutrient composition of the diets was calculated to cover the nutrient requirements of the birds throughout the study (Table A1). The birds had free access to water. Fecal samples were taken daily at the same time on days 1–7, and on days 10, 14, 21, 28, and 35. For each sampling day, a plastic cover was placed on the floor of the pen on top of the litter for 1 hr. Five separate fresh fecal droppings were collected, excluding cecal droppings from the sheet, and stored individually at −80°C within 1 hr of collection for further analyses.

### 2.2 DNA extraction and 16S rRNA sequencing

DNA was extracted from 0.2 g of feces using the Qiagen QIAamp Fast DNA stool mini kit (Qiagen, Hilden, Germany), according to the manufacturer's instructions with an extra bead‐beating step, and eluted in 50 μl. Extracts were checked on a 2200 Tapestation (Agilent Technologies, Santa Clara, CA).

16S rRNA gene amplicon sequences were used to monitor bacterial community composition in the developing chicks’ feces. The V3‐4 region of the 16S rRNA gene was amplified by PCR using the primers CVI_V3‐forw CCTACGGGAGGCAGCAG and CVI_V4‐rev GGACTACHVGGGTWTCT with the following amplification conditions: 98°C for 2 m, followed by 15 cycles of 98°C for 10 s, 55°C for 30 s, and 72°C for 10 s, and finally by 72°C for 7 min. PCR products were checked with gel electrophoresis, and sequencing was performed using a MiSeq sequencer (Illumina Inc., San Diego, CA).

### 2.3 Sequence processing and statistical analyses

All sequence processing and statistical analyses were performed in R 3.4.0 (R Core Team, 2014). The 16S rRNA gene sequencing reads were filtered, trimmed, dereplicated, chimera‐checked, and merged using the dada2 package (v.1.4.0; Callahan et al., 2016) using standard parameters (*TruncLength *= 240,210) and reads were assigned with the SILVA v.132 classifier (Quast et al., 2012). Downstream analyses were performed with the phyloseq (McMurdie & Holmes, 2013) and vegan (Oksanen et al., 2007) packages. Good's coverage was >0.999. Prior to analyses, the data were rarefied to 28,523 reads per sample (*rarefy_even_depth, seed=1*). The final dataset contained 1,475 species variants (SVs). Sequences are deposited in NCBI's Sequence Read Archive under BioProject accession number PRJNA517082.

The number of SVs per sample was used as a measure of observed richness. Patterns in richness (α‐diversity) over time were evaluated with a linear regression. Abundances in taxa over time were reported throughout the manuscript as mean ± *SD*. To evaluate changes in community structure over time (β‐diversity), we performed a Principal Coordinates Analysis (PCoA) of Bray–Curtis distances between samples, and clustering between samples was assessed using *adonis*. Dispersion of samples was evaluated using *betadisper*. To identify taxa with consistently varying abundances over time, we performed an ANOVA for the effect of sampling time on the abundance of each genus, selected genera for which *p* < 0.001, standardized them according to their relative temporal abundance patterns (Shade, McManus, & Handelsman, 2013), and clustered them using Euclidean distances and Ward's method.

## 3 RESULTS

### 3.1 Bacterial abundance and α‐diversity

**Figure 1:**
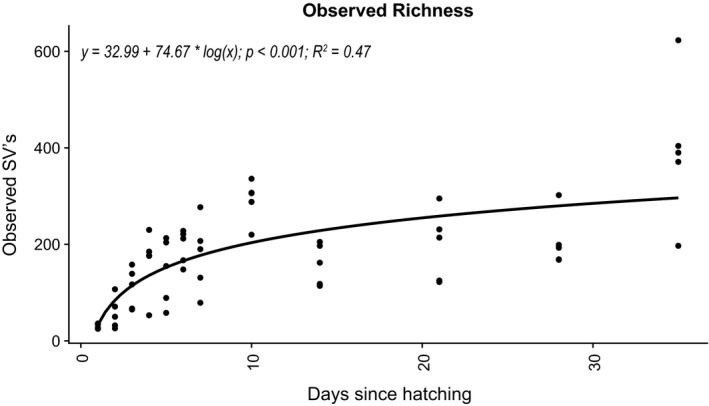
Microbial richness in feces over time. A single linear regression of observed diversity over time. The derived formula, *R* ^2^ and significance are displayed above. SV: species variant

To characterize the microbial community of the developing chicks’ fecal microbiomes, we first explored community diversity. For the period studied, observed richness increased from an average of 31.4 ± 5.0 SVs on day 1 to 397 ± 155.5 SVs on day 35. Age of the bird significantly predicted the number of observed SVs per sample with the formula diversity = 32.99 + 74.67 × log(time), *R* ^2^ = 0.47, *p* < 0.001 (Figure 1).

**Figure A1:**
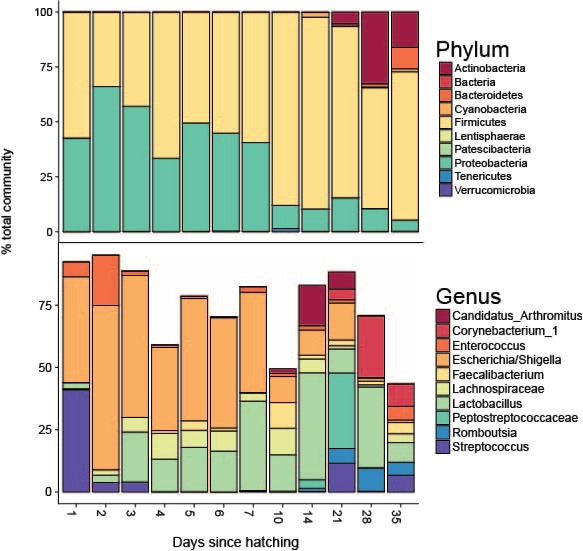
Relative abundances of 10 most abundant phyla (top) and genera (bottom) over time. Average values per time are displayed

Throughout the experiment, the fecal microbiome gradually shifted from a predominance of Proteobacteria to Firmicutes (Figure A1). On day 1, the community was dominated by *Escherichia/Shigella* (42.5 ± 2.5% of the community on average) and *Streptococcus* (41.1 ± 3.1% on average). While *Streptococcus* decreased to an average of 0.1 ± 0.1% by day 4, *Escherichia/Shigella* decreased much more gradually, falling to similar levels as *Streptococcus* by day 28. In contrast, *Lactobacillus*, which accounted for an average of 0.2 ± 0.2% of the community on day 1, gradually increased to account for 42.9 ± 34.0% by day 14, fluctuating thereafter (Figure A1). On days 14 and 21, Bacteroidetes and Actinobacteria increased to above 1% of the community, respectively.

### 3.2 β‐diversity

**Figure 2:**
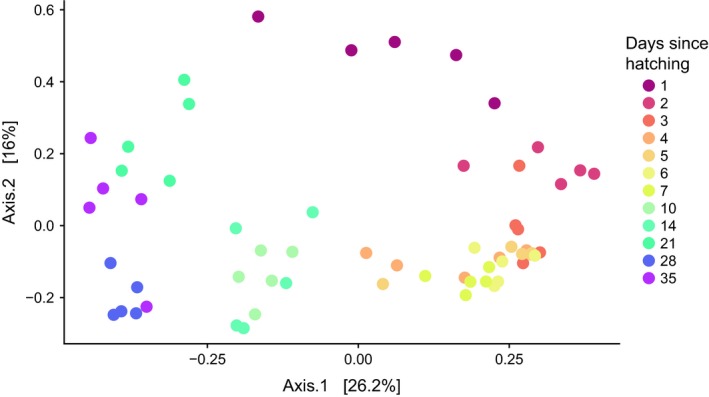
Change in community composition of broiler fecal microbiomes over time. A Principal Coordinates Analysis plot of Bray–Curtis distances between samples

We evaluated changes in community composition with a PCoA of Bray–Curtis distances. Samples clustered significantly according to time (adonis, pseudo *F* = 6.98, *R* ^2^ = 0.61, *p* < 0.001), increasingly diverging from day 1 samples (Figure 2). While variability between replicates did not change significantly over time (ANOVA test on homogeneity of group dispersions, *p* = 0.10), a *t* test examining the distances within temporal replicates between samples taken from days 1–7 and from days 10–35 found that heterogeneity between samples was significantly higher (*p* = 0.006) in the later stages, indicating increased heterogeneity over time.

### 3.3 Temporal dynamics

**Figure 3:**
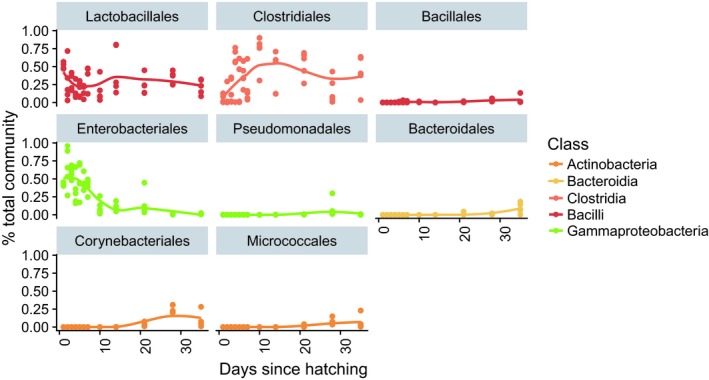
Order‐specific colonization pattern over time. The eight most abundant taxonomic orders colored according to their corresponding classes, with fitted LOWESS curves. The taxa displayed account for 98.7 ± 2.2% of the community across all samples, on average

In order to examine temporal dynamics, we selected the eight most abundant bacterial orders among all fecal samples collected in the study, which accounted for 98.7 ± 2.2% of the community on average throughout the entire experiment (Figure 3). These orders showed unique temporal patterns: Lactobacilliales were highest during the first day after hatching (50.4 ± 4.3% of the community), and decreased in relative abundance until day 5, remaining at an average of 25.7 ± 9.9% of the community for all subsequent time points. Similarly, Enterobacteriales was highest during day 1 (42.6 ± 2.5%) but decreased gradually until day 14, remaining at 5.7 ± 7.9% on average until day 35. In contrast, members of Clostridiales grew from 6.8 ± 5.7% of the community on day 1 to 70.9 ± 17.7% on day 10, and maintained an average relative abundance of 38.4 ± 15.5% on average until day 35. Corynebacteriales were rare or undetectable (<0.01% of the community) until day 14, but made up 25 ± 5.4% of the community on day 28, remaining above 1% of the total community between days 21 and 35. Bacillales, Pseudomonadales, Bacteroidales, and Micrococcales gradually increased between days 21 and 35, but collectively remained below 10% of the total community for all samples.

**Figure 4:**
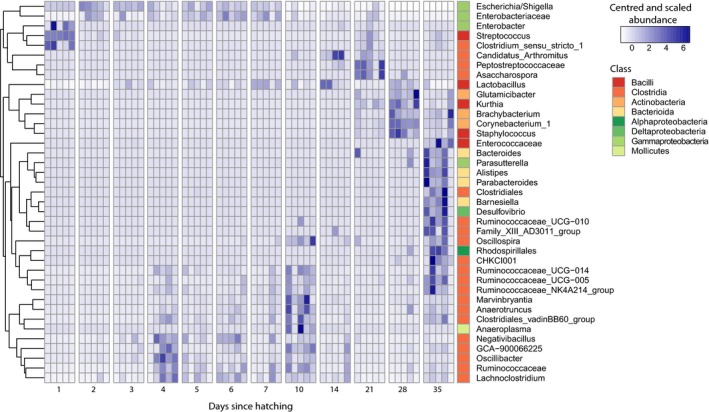
Core dynamic taxa of the developing broilers’ fecal microbiome. Heatmap of the abundance of 39 genera exhibiting significant (ANOVA, *p *< 0.001) temporal dynamics. These taxa represented 72.9 ± 18.9% of the total community of all samples, on average. Abundances were standardized by time, per genus, and genera were clustered according to the resulting temporal response patterns. Class membership is displayed on the right column

To further explore genera which showed consistent dynamics over time, we performed an ANOVA to compare the relative abundances of each genus at each time point, and selected taxa for which *p* < 0.001 for further analysis. The 39 resulting taxa were distributed among the phyla Actinobacteria, Bacteroidetes, Firmicutes, Proteobacteria, and Tenericutes, and represented an average of 72.9 ± 18.9% of the total community (Figure 4). These genera exhibited significant changes in relative abundance over time. On day 1, *Streptococcus*,* Clostridium* sensu stricto, and *Enterobacter* exhibited significantly higher abundances than on any other sampling day. An unclassified genus belonging to *Enterobacteriaceae* and *Escherichia/Shigella* maintained significantly higher abundances between days 3 and 7 after hatching, while a large group of genera predominantly belonging to Clostridia and including several members of *Ruminococcaceae* exhibited higher abundances between days 4 and 10. In contrast, the relative abundance of *Candidatus Arthromitus* was highest on days 14 and 21; *Asaccharospora* and a *Peptostreptococcaceae* SV were highest on day 21; a group of Bacilli and Actinobacteria including *Lactobacillus*,* Glutamicibater*, and *Corynebacterium* were highest on day 28, and a large and diverse group of bacteria including *Alistipes*,* Barnesiella*, and *Oscillospira* were most abundant on day 35.

## 4 DISCUSSION

A better understanding of successional patterns in the developing broiler GIT microbiome has the potential to improve microbiome management practices and prevent disease in poultry (Kogut & Arsenault, 2016). We studied the fecal microbiome of developing broilers with an emphasis on the first week after hatching. As expected, community richness increased rapidly over time, stabilizing by day 14. This is in accordance with other studies monitoring the development of the cecal microbiomes of broilers over time (Ballou et al., 2016; Oakley, Buhr, et al., 2014; Oakley & Kogut, 2016); however, the patterns observed during the first half of our experiment further show that the increase in diversity is highest during the first 7 days and is best represented by a linear regression with the logarithm of time as the explanatory variable, highlighting the importance of the first week after hatching in the development of the GIT microbiome in broilers (Nurmi & Rantala, 1973).

The fecal microbiomes exhibited community dynamics which were consistent between temporal replicates over time. Notably, the bacterial communities were less variable between replicates during the first week than in samples taken at later time (i.e., Days 10–35), in sharp contrast to previous findings in a T‐RFLP of developing broilers’ ceca (Crhanova et al., 2011). In our case, the increase in between‐sample variability was positively correlated with the increase in richness (data not shown), and is likely a result of the increasing complexity of the community.

Several reports of microbial colonization of the developing chicken GIT state that maximum microbial loads in both the cecum and ileum are reached within the first 5 days after hatching, and remain at these levels thereafter (Apajalahti, Kettunen, & Graham, 2004; Van Der Wielen, Keuzenkamp, Lipman, Van Knapen, & Biesterveld, 2002). In our experiment, the rate of increase in community richness subsided after day 10, but the community composition continued to change, suggesting that competition between microbes and host responses continued to alter dominance patterns within the community even after it was fully colonized.

Indeed, the patterns observed in the eight dominant orders in the community further suggest the presence of successional dynamics in three stages. On the first day of the experiment, approximately 24 hr after hatching, the community was dominated by the orders Enterobacteriales and Lactobacillales, consisting mostly of *Escherichia/Shigella* and *Streptococcus* SVs. *Escherichia/Shigella* is considered a putative pathogen, and is generally found in higher proportions in broiler feces than in cecal samples (Oakley, Lillehoj, et al., 2014). Dominance of *Escherichia/Shigella* was previously reported in the fecal microbiomes broilers 3 days after hatching, which was no longer present the following day (Sekelja et al., 2012), as well as in cecal samples (Ballou et al., 2016). In our study, the prevalence of *Escherichia/Shigella* decreased gradually between days 1 and 7. *Streptococcus*, which accounted for 41% of the community on day 1 decreased sharply to 3% on the following day. It is possible that these taxa were vertically transmitted in ovo, as previously reported for *Escherichia/Shigella* (Pedroso & Lee, 2015).

In the second stage, the gradual displacement of Enterobacteriales and Lactobacillales from the broiler feces was accompanied by an increase in the dominance of the order Clostridiales, starting on day 3 and represented mostly by *Ruminococcus*‐related SVs and members of *Lachnospiraceae*. The surge in *Ruminococcus*‐related SVs at 1 week after hatching has previously been reported in broiler ceca (Ballou et al., 2016; Oakley, Buhr, et al., 2014), and the abundance of *Ruminococcus* has been positively correlated with the expression of IL1β and IL6 two pro‐inflammatory cytokines, 6 weeks after hatch (Oakley & Kogut, 2016). Our results show that colonization by Clostridiales begins as early as 4 days after hatching.

In the third stage, this order remained dominant but we detected the partial displacement of Clostridiales after day 10 by a diverse cluster of common gut‐associated bacteria including members of Corynebacteriales, Bacilliales, Pseudomonadales, Micrococcales, and Bacteroidales. Several of the individual SVs which exhibited their highest abundance during the third stage have been shown to be beneficial gut microbes: *Lactobacillus* has been detected in the small intestine of 21‐day‐old broilers (Pedroso & Lee, 2015), and is routinely administered in probiotic treatments or stimulated by prebiotic treatments (Ballou et al., 2016; Pedroso & Lee, 2015; Ricke, 2015); and *Candidatus Arthromitus* has been associated with the development of gut innate and adaptive immune functions in mice, specifically in the ileum (Bolotin et al., 2014).

The observed temporal patterns show successional dynamics in the fecal microbiome of developing broilers in three stages, with radical changes in community composition on days 3–4 and 3 weeks after hatching. These changes may be a result of host‐microbiome interactions arising from the development of the host's immune or enzymatic potential, which have been shown to drastically change during the first 14 days after hatching (Sell, 1995; Sklan, 2001). For example, immunoglobulins can be detected in the cecum approximately 10 days after hatching (Matulova et al., 2013; Van Immerseel et al., 2002). The relationship between the host's immune development and its resident microbiota has been previously reported for chickens (Volf et al., 2016). In one case, Crhanova and colleagues reported a decrease in the expression of β‐defensins in broiler ceca on the third day after hatching and an increase in the expression of IL‐8 and IL‐17 1 day later (Crhanova et al., 2011). It must be noted, however, that while the change in immune response reported by Crhanova et al. was rapid, the decrease in the relative abundance of Enterobacteriales and Lactobacillales was gradual. Alternatively, interactions between microbes may have played a role in modulating community assembly through competitive exclusion. We propose that resource competition may explain the successional shifts observed, as taxa which were initially present through vertical transmission or early colonization (i.e., Lactobacillales and Enterobacteriales) were displaced within the first week after hatching by rapid‐growing members of Clostridiales, whose abundance was in turn limited by the influx of diverse, specialist taxa associated with the adult broiler microbiome. Competition for resources has been previously proposed as a key component of microbial community assembly in various environments (Ho, Lüke, Reim, & Frenzel, 2016), and the temporal pattern of succession matches that observed in soils (Jurburg et al., 2017), suggesting that it may be related to the growth rates of various microbial community members. The measurement of resource availability in the GIT may aid in revealing the role of resource competition in successional dynamics in the broiler GIT.

Our findings support the notion that early, transient colonizers in the broiler GIT may greatly influence the adult microbiome (Ballou et al., 2016). Several studies have showed that the freshly hatched chicken is susceptible to colonization by a wide range of microbes (Polansky et al., 2016; Volf et al., 2016). In particular, one study found that the cecal microbiomes of freshly hatched chickens inoculated with the cecal microbiomes of donor chickens of different ages underwent less community shifts during development the older the donor chickens were (Volf et al., 2016). Further research should examine whether successional patterns in the chicken gut depend on the composition and functional profile of the initial inoculum.

Our findings also align with the idea that first week after hatching is critical to broiler microbiome development (Nurmi & Rantala, 1973), and allow us to identify successional stages in the fecal microbial community, opening the black box which has been the first week of broiler microbiome development. The boundaries of these successional stages (Days 3–4 and 10–14 after hatching) are likely the optimal times for microbiome interventions, as it is during this time that competitive dynamics are likely to be the strongest and most susceptible to change. Our experiment spanned the average lifetime of commercial broilers (35 days), which is considerably shorter than that of chickens in other environments. Had our experiment lasted longer, it is likely that we would have detected further changes in the fecal microbiome, as our chickens would have continued to physiologically develop. For example, a study of egg‐laying hens over 14 months observed large, consistent shifts in their cecal microbiomes 2 and 6 months after hatching (Videnska, Sedlar, Lukac, & Faldynova, 2014). Similarly to our study, the authors note the dominance of the phyla Proteobacteria and Firmicutes, particularly belonging to *Escherichia* and *Lachnospiraceae* during the first week after hatching (Videnska et al., 2014).

We sampled the fecal microbiome because our daily sampling scheme called for a rapid sampling methodology, and because we were interested in temporal dynamics rather than in the emergence of specific pathogens. Whether the fecal microbiome is representative of the GIT microbiome is the subject of debate: the fecal microbiome is considered a proxy for the composition of the small intestine; it has been found that the fecal microbiome is more variable than the cecal microbiome and exhibits higher proportions of Enterobacteriales and Lactobacillales (Oakley & Kogut, 2016; Oakley, Lillehoj, et al., 2014; Sekelja et al., 2012). Nevertheless, our results show temporally consistent patterns with age, and detected taxa which are commonly present in different segments of the GIT. It is likely that cecal samples would have displayed lower between‐replicate variability, and further study of the correspondence between temporal dynamics in the fecal and cecal microbiomes is warranted. Furthermore, while our study assessed the community assembly of the broiler fecal microbiome in a controlled environment, the influence real‐world sources of variation such as seasonality, farm, and feed on the development of the chicken microbiome remains to be examined.

## CONFLICT OF INTERESTS

None declared.

## AUTHORS CONTRIBUTION

S.D.J. conceived of the experiment, performed the analyses and wrote the manuscript; A.J.M.J. provided the samples; M.S.M.B., D.C., J.v.d.G., and A.B. aided with writing and data interpretation.

## ETHICS STATEMENT

Ethical approval for sampling was not required, as fresh fecal droppings were collected noninvasively.

## Data Availability

Sequences are deposited in NCBI's Sequence Read Archive under BioProject accession number PRJNA517082.

## APPENDIX 1

### 1.1

**Table A1:** Ingredient and nutrient composition of the experimental diets

	Starter Days 0–14	Grower Days 14–28	Finisher Days 28–42
%			
Maize	57.9	57.6	59.8
Soyabean meal	32.0	33.8	32.7
Maizegluten meal	2.7	0.0	0.0
Palm oil	2.0	2.5	3.0
Soya oil	0.7	2.3	1.6
Chalk	1.6	1.3	1.0
Mono calcium phosphate	1.7	1.3	0.8
NaCl	0.2	0.3	0.3
Sodium bicarbonate	0.2	0.1	0.1
Premix	0.5	0.5	0.5
L‐lysine HCl	0.2	0.1	0.0
DL‐methionine	0.2	0.2	0.2
L‐threonine	0.1	0.0	0.0
g/kg			
Dry matter	882	883	881
Ash	65	59	51
Crude protein	224	215	210
Fat	63	82	82
Crude fiber	24	25	25
Starch	360	354	367
Sugars	40	42	41
NSP	137	139	139
ME broilers (MJ/kg)	12.10	12.50	12.60
